# Effectiveness of the Malnutrition eLearning Course for Global Capacity Building in the Management of Malnutrition: Cross-Country Interrupted Time-Series Study

**DOI:** 10.2196/10396

**Published:** 2018-10-03

**Authors:** Sunhea Choi, Ho Ming Yuen, Reginald Annan, Trevor Pickup, Andy Pulman, Michele Monroy-Valle, Nana Esi Linda Aduku, Samuel Kyei-Boateng, Carmen Isabel Velásquez Monzón, Carmen Elisa Portillo Sermeño, Andrew Penn, Ann Ashworth, Alan A Jackson

**Affiliations:** 1 Human Development and Health Faculty of Medicine University of Southampton Southampton United Kingdom; 2 Faculty of Medicine University of Southampton Southampton United Kingdom; 3 Department of Biochemistry and Biotechnology Kwame Nkrumah University of Science and Technology Kumasi Ghana; 4 Facultad de Ciencias de la Salud Universidad Rafael Landívar Ciudad de Guatemala Guatemala; 5 Department of Nutrition and Diets Hospital Nacional “Dr Juan José Fernandez” Zacamil San Salvador El Salvador; 6 Department of Population Health London School of Hygiene and Tropical Medicine London United Kingdom

**Keywords:** eLearning, severe acute malnutrition, WHO guidelines for malnutrition, capacity building, staff development, quality improvement, nutrition training and education

## Abstract

**Background:**

Scaling up improved management of severe acute malnutrition has been identified as the nutrition intervention with the largest potential to reduce child mortality, but lack of operational capacity at all levels of the health system constrains scale-up. We therefore developed an interactive malnutrition eLearning course that is accessible at scale to build capacity of the health sector workforce to manage severely malnourished children according to the guidelines of the World Health Organization.

**Objective:**

The aim of this study was to test whether the malnutrition eLearning course improves knowledge and skills of in-service and preservice health professionals in managing children with severe acute malnutrition and enables them to apply the gained knowledge and skills in patient care.

**Methods:**

This 2-year prospective, longitudinal, cross-country, interrupted time-series study took place in Ghana, Guatemala, El Salvador, and Colombia between January 2015 and February 2017. A subset of 354 in-service health personnel from 12 hospitals and 2 Ministries of Health, 703 preservice trainees from 9 academic institutions, and 204 online users participated. Knowledge gained after training and retention over time was measured through pre- and postassessments comprising questions pertaining to screening, diagnosis, pathophysiology and treatment, and prevention of malnutrition. Comprehension, application, and integration of knowledge were tested. Changes in perception, confidence, and clinical practice were assessed through questionnaires and interviews.

**Results:**

Before the course, awareness of the World Health Organization guidelines was 36.73% (389/1059) overall, and 26.3% (94/358) among in-service professionals. The mean score gain in knowledge after access to the course in 606 participants who had pre- and postassessment data was 11.8 (95% CI 10.8-12.9; *P*<.001)—a relative increase of 41.5%. The proportion of participants who achieved a score above the pass mark posttraining was 58.7% (356/606), compared with 18.2% (110/606) in pretraining. Of the in-service professionals, 85.9% (128/149) reported applying their knowledge by changing their clinical practice in screening, assessment, diagnosis, and management. This group demonstrated significantly increased retained knowledge 6 months after training (mean difference [SD] from preassessment of 12.1 [11.8]), retaining 65.8% (12.1/18.4) of gained knowledge from the training. Changes in the management of malnutrition were reported by trained participants, and institutional, operational, and policy changes were also found.

**Conclusions:**

The malnutrition eLearning course improved knowledge, understanding, and skills of health professionals in the diagnosis and management of children with severe acute malnutrition, and changes in clinical practice and confidence were reported following the completion of the course.

## Introduction

### Background

Undernutrition (low weight-for-age) is associated with 3.1 million child deaths annually [1]. In the 2013 Lancet Series, scaling up the management of severe acute malnutrition (SAM, ie, severe wasting, severe wasting with edema, or edema) was identified as the nutrition intervention with the largest potential to reduce child mortality [2]. Lack of operational capacity at all levels of the health sector, however, constrains scale-up [3]. In countries most affected by SAM, training and curricula are outdated or nonexistent and misaligned with strategic and operational needs [4], leading to a workforce that is ill equipped to identify and treat malnourished children [5]. This limited knowledge and competency of health professionals in managing children with SAM led the International Pediatric Association to pass a resolution in 2010, stating that all pediatricians and related health professionals should have the identification and treatment of severe malnutrition as a core competency [6]. It was against this background that the International Malnutrition Task Force of the International Union of Nutritional Sciences joined with the University of Southampton to develop an eLearning training course on “Caring for infants and young children with severe malnutrition” that could be accessed at scale through the internet (Multimedia Appendix 1). The project goal was to build the capacity of the health sector workforce globally to manage SAM and reduce child deaths. The rapid spread of the internet and access to information technology (IT) across the developing world provides exciting new opportunities for delivering training in a way that was not possible before. If utilized effectively, we believe eLearning can make a significant contribution to building capacity for improved malnutrition management, supporting the aims of the Scaling Up Nutrition Movement, through a high-volume training of relevant personnel around the world.

The World Health Organization (WHO) guidelines for the management of SAM have been shown to be demonstrably effective when implemented appropriately [2,7-9], and well-trained, motivated staff have been shown to reduce SAM mortality in practice [9-11]. It has been suggested that by harnessing the potential of technology, particularly the internet, it should be possible to train health professionals at scale [12-14].

eLearning is at least as effective as traditional teaching in facilitating knowledge and skills learning [15-17], and it can be cost effective [18]. Learning is an active process of constructing knowledge (constructivist view) [19,20], and instruction is a process of supporting that construction [21]. Our course offers scenario-based eLearning, which aims to provide contextualized learning of patient care through malnutrition cases and their management. It creates a learning environment that promotes *deep* rather than *surface* learning [22-26], in which learners can actively construct new and meaningful knowledge [27-29]. Such an environment enables the learner to see the *relevance* of the new learning content in their context and situation and relate it to their existing knowledge or experience. The course incorporates aspects of problem-based learning [30] and situated learning with authentic tasks [31]. These approaches aim to facilitate active learning with contextually relevant tasks through which the learner can acquire, integrate, and apply knowledge and skills and subsequently be able to apply the practiced knowledge and skills in their clinical work. An example is virtual patients, which were created to facilitate contextualized learning of basic and clinical sciences for early medical training [32] and case-based learning through branching scenarios for clinical reasoning and decision-making skills training [33-37]. Adaptive learning aims to provide tailored training to suit the individual needs of the learner to maximize performance, and it is hypothesized that the greater the degree of adaptation and the inclusion of content adaptation, the greater is its effectiveness [38]. Factors such as learners’ knowledge levels, difficulty level of learning content, characteristics of the learner (learning styles, cognitive styles), and their preferences are used in adaptive eLearning systems to customize the content, navigation, presentation (media and layout), and materials/tools [38,39].

Despite widespread use, there are important gaps in knowledge regarding eLearning. For example, little is known about the effectiveness of eLearning on patient outcomes [16], and the environments in which eLearning occurs tend to be limited. For example, in a systematic review of undergraduate health professional education commissioned by the WHO, only 5 of 49 eLearning studies were from low- or middle-income countries (Brazil, China, and Thailand), and only 14 considered professions allied to medicine such as nursing [15]. The authors of the review recommended that future research should assess the outcomes of eLearning in health care training (1) in low- and middle-income countries and (2) among professionals allied to medicine. They also recommended evaluating the impact of eLearning on long-term retention of knowledge and skills. Our study has relevance with each of these knowledge gaps.

### Study and Objectives

Since its launch, the malnutrition eLearning course has been used by over 14,000 health professionals, trainees, and educators, and positive anecdotal feedback has been received [40]. Knowledge scores embedded in each module show improvement on completion of the modules; however, although they are encouraging, they do not adequately show whether the knowledge gained is actually applied in practice and whether this leads to improved management of severely malnourished children. This evaluation was therefore undertaken to investigate whether (1) the malnutrition eLearning course improves the knowledge and skills in managing children with SAM of in-service and preservice health professionals (*think differently*) and enables them to apply the gained knowledge and skills in patient care (*act differently*) and (2) their application of the gained knowledge and skills leads to improved clinical practice and outcomes for severely malnourished children in resource-poor countries. In this paper, we address the first hypothesis. The second hypothesis is addressed in a separate paper.

## Methods

### Design

This is a prospective, longitudinal, cross-country, interrupted time-series study investigating the effectiveness of an educational intervention in facilitating the gain of knowledge and skills and changes in clinical practice, which could lead to improved care of children with SAM. Kirkpatrick training evaluation model [41] was applied to the study design. It comprises 4 levels: reaction, learning, behavior, and results.

### Intervention

#### Course Developers

The eLearning course was developed by members of the International Malnutrition Task Force and the University of Southampton. The content was prepared mainly by RA and AA, and the interactive, task-based design was created by SC. The host platform and course were developed by SC and the University of Southampton Faculty of Medicine eLearning team. For many years, AA and AAJ served as advisers to the WHO on inpatient management of severe malnutrition and on training of health professionals. AA also served as a facilitator for WHO regional training courses to improve the inpatient management of severe malnutrition.

#### Development Process

The design of the course is scenario-based and interactive to actively support and engage the user in the learning process. The course uses a range of rich media; however, it can be run on a low specification computer with a limited internet speed. In addition, a stand-alone CD version of the course supports the areas where internet access is not possible. The content is set at a level suitable for the primary target groups, namely in-service and preservice health professionals who are or will be working with undernourished children. Secondary target groups are educators and trainers in medical, nursing, and health science schools and organizations with responsibility for preservice training. The target regions are those where the prevalence of malnutrition is high, notably sub-Saharan Africa, India and the subcontinent, and Latin America [42]. Upon the completion of the course development in 2010, a field test (beta testing) was conducted in Uganda [43] (Multimedia Appendix 2) to assess the effectiveness of the course and the appropriateness of its delivery for the target users and regions. Overall, 86 participants, including doctors, medical students, nurses, midwives, and nutritionists, participated in the study, and the results indicated that the course design supports learning for different health professionals, and the course is accessible with limited internet bandwidth.

#### Revisions and Updates

After the field test in Uganda, adjustments were made to eliminate ambiguities and ensure smooth functioning of the course. In 2014, the content was slightly revised to match the updated WHO case-management guidelines. No further changes were made either before or during the study. Currently, the course is being reimplemented from Adobe Flash to html5, and the new version will be made freely available in October 2018.

#### Quality Assurance

The course content conforms to the WHO case-management guidelines [44-46]. Standard anthropometric techniques are used for assessing nutritional status. The accuracy of the content was checked by 3 experienced public health nutritionists working independently.

#### Replicability

The course is freely available from the University of Southampton nutrition portal [47]. The design solution has been disseminated through presentations, poster, and handouts at eLearning events (exhibitions and conferences) held by the eLearning team in 2009 and 2011. The dissemination materials are available from the medicine eLearning website [48]. The technical solutions designed for the nutrition portal and course implementation are available in published papers [49,50].

#### Access

There are no preconditions and anyone can access the course. Study participants gained access by registering for the course in the University of Southampton nutrition portal, either via their own laptop or from 1 of their institution’s computers, or by using a CD version.

#### Mode of Delivery and Content

The course offers interactive learning in 3 modules: Module 1: Definition and classification of malnutrition; Module 2: How to identify children with malnutrition; and Module 3: How to manage children with malnutrition. The course facilitates learning on how to differentiate between chronic and acute malnutrition; pathophysiological changes in malnutrition and implications for treatment; how to assess and screen children for malnutrition and interpret the results for action; how to manage malnourished children using the WHO Ten Steps; hospital compared with community-based management; the importance of an integrated approach between hospital and community; and how to support mothers and caregivers to prevent the recurrence of malnutrition.

**Figure 1:**
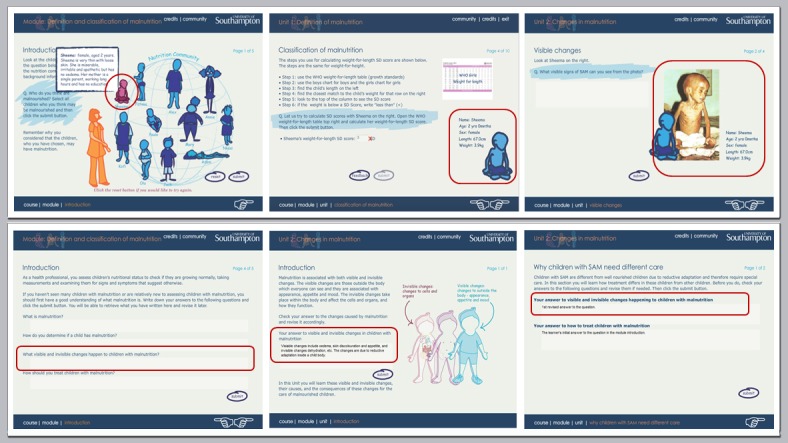
Overarching design strategies: illustrative snapshots of scenario and task-based activities (top row) and revisable reflective questions (bottom row). The top row shows the introduction of malnutrition concepts and classification leading to practical application through Sheema, and the bottom row shows a reflective question, "What visible and invisible changes happen to children with malnutrition?", appearing at 3 different learning points.

The course is designed based on a constructivist view of learning—“an active process of constructing knowledge” [19-21]—and aims to facilitate the activities required for learning to occur, which are apprehending structure, integration, application, and reflection [29,51-53]. The 2 key overarching design strategies are (1) scenario- and task-based learning activities using authentic cases to support an interactive process of acquiring, linking, integration, and application of knowledge and skills and (2) activities embedded within the content, that is, revisable reflective questions and conversational style to promote active reflection and cognitive dialogue. Figure 1 shows illustrative snapshots of the 2 design strategies.

Each module usually takes 2 to 3 hours to complete but is asynchronous as users take the course in their own time. Each module comprises several interlinked subunits, and users can track their progress through a set of multiple-choice questions at the beginning and end of each module. Users can navigate within the course as desired.

#### Human Involvement and Prompts

No support or assistance was given to participants while they were taking the course. The participants were introduced to the course at the end of the prestudy data collection and asked to complete the course within 3 weeks. There were no cointerventions or prompts.

### Participants and Setting

A subset of target course users and geographic locations was selected for the study that was conducted face-to-face in Ghana and in Guatemala, El Salvador, and Colombia (grouped for convenience as Latin America) and also globally online, between January 2015 and February 2017. Study participation was voluntary. The study consisted of 2 groups: (1) in-service health professionals and preservice trainees studying health science subjects in Ghana and Latin America (center-based group) and (2) a self-selected group of global users who elected to take the course during a defined period of time (remote learning group).

#### Center-Based Group

Health care and academic institutions in Ghana (Ashanti region) and Latin America (El Salvador, Guatemala, and Colombia) were invited to participate. Health care institutions providing pediatric care (hospitals and community health centers) and academic institutions offering health science training programs (universities and training colleges) were eligible to participate.

In Ghana, 10 hospitals and their linked community health centers, and 8 academic institutions were contacted. Of these, 9 hospitals (and linked health centers) and 7 academic institutions accepted the invitation and participated in the evaluation. In Latin America, 3 of the 4 contacted hospitals, 2 of the 8 academic institutions, and the Ministries of Health for Guatemala and El Salvador accepted the invitation. Educators at the participating academic institutions were invited to 1 of the 3 2-day workshops during which they were introduced to the malnutrition eLearning course, and they developed action plans to initiate the course at their home institutions. All participants in Latin America and preservice participants in Ghana were introduced to the course by the research team or trained educators at their institutions.

They were then given 3 weeks to complete the course either online or using the CD version installed on their institutional computers. For the Ghana in-service participants, a 2-day, self-directed training session was organized at each participating hospital and facilitated by the research team.

#### Remote Learning Group

Between October 2015 and January 2016, new users of the malnutrition eLearning course were invited at the time of enrollment to take part in the study. The inclusion criteria were that they were (1) in-service health professionals who worked closely with children or (2) preservice health professionals studying medicine or allied health science subjects and were not part of the participating institutions in Ghana and Latin America. Of the 322 who responded, 263 met the inclusion criteria. They came from 38 health care, academic, and nongovernment organizations across 40 countries.

Baseline (pre) data were collected before the modules were taken and follow-up data were captured at 3 time points over the period of 1 year: post (immediately after), 6 months, and 12 months after the training.

### Ethical Approval

This study was reviewed and approved by the ethics committees of the University of Southampton, United Kingdom (Ethics ID:12872), Komfo Anokye Teaching Hospital and Kwame Nkrumah University of Science and Technology, Ghana, and the Universidad Rafael Landívar, Guatemala. Informed consent was obtained from participants and institutions before the commencement of the study.

### Data Collection

The effectiveness of the malnutrition eLearning course for learning was evaluated using a mixture of quantitative and qualitative methods comprising assessments, questionnaires, and interviews and focus groups with individual participants.

#### Assessments (Pre, Post, and 6 Months)

To measure the participants’ gain in knowledge from the course immediately after the training and retention of the gained knowledge 6 months later, 2 sets of comparable assessments were prepared for the baseline and follow-up phases. One assessment was assigned for the prestudy and the other for the post and 6-month follow-up studies. Each consisted of 32 questions on key topics identified from the course, and the questions were prepared to test comprehension, application, and integration of knowledge. Participants ranged from medical doctors to community health workers, and differences in existing knowledge between professional groups as well as within each group were anticipated. A standard setting procedure using the Ebel method [54], which allows an expected standard to be set in the form of a pass mark based on the difficulty and importance of each question, was carried out for each assessment. The pass marks established for the pre- and follow-up assessments were 37.4% and 36.4%, respectively.

For the center-based group, assessments were conducted *exam-style* within a set time under supervision by members of the research team. The total score for each assessment was calculated based on each question carrying the same weight. For the remote learning group, equivalent online versions of the assessments were prepared. To minimize any carryover effect between assessments, scores were released to participants only after the final 6-month assessment.

The assessment scores were analyzed to determine (1) participants’ acquisition of core knowledge and skills from the training, (2) how many and who acquired the pass marks, and (3) retained knowledge at 6-month follow-up. Key influencing factors considered were profession, prior training in the management of SAM, adherence to the WHO guidelines at baseline, partial or full completion of the malnutrition eLearning course, and application of knowledge in the follow-up period.

#### Questionnaires (Pre, Post, 6 Months, and 12 Months)

Questionnaires, consisting of closed and open-ended questions, were administered at 4 time points (pre, post, 6 months, and 12 months) with a combination of similar (to investigate changes over time) and time-specific questions. These questionnaires explored whether participants completed the course, perceived changes in knowledge and understanding of malnutrition and its management, application of knowledge and associated outcomes, confidence in performing relevant tasks, changes in clinical practice made by in-service participants and resulting policy changes at their health care facilities, and benefits gained from the training. These questionnaires were administered face-to-face for the center-based group and online for the remote learning group.

#### Interviews (Pre, Post, 6 Months, and 12 Months) and Focus Groups (Pre and 12 Months)

**Figure 2:**
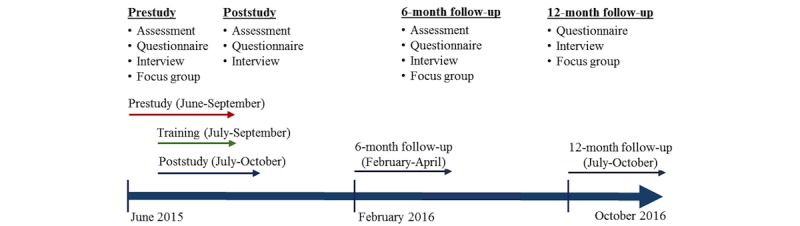
Study timeline and data collection methods used.

Semistructured interviews were conducted with the participants who volunteered to share further details of their experience, namely what they gained from the course, if and where they applied their gained knowledge and associated outcomes, if and how their perceptions about malnutrition and its management changed, and changes in the management of SAM made by them and observed at their workplaces. The interviews were audio-recorded and transcribed verbatim. Focus groups were conducted in Latin America with similar aims and structure to the interviews. Each consisted of 3 to 6 participants and was audio-recorded and transcribed verbatim. Figure 2 summarizes the data collection methods used.

### Relation Between Knowledge Gain, Application, and Confidence in Patient Care

Improvement in knowledge and understanding was reported in 9 topic areas, from “types and classification of malnutrition” to “inpatient therapeutic care,” and the scores were aggregated and averaged to represent the overall response. Improvement in confidence in performing tasks was reported in 6 areas, from “screening children for malnutrition” to “managing children with severe acute malnutrition using WHO Ten Steps,” and the scores were aggregated and averaged to represent the overall response. Participants’ gain in knowledge, its application in patient care, and reported increase in confidence from the training and subsequent application of the gained knowledge were assessed over time.

Our hypothesis was that the relation between improvement in knowledge and confidence would be weak at postassessment but would improve positively over time through the application of knowledge and experience of positive outcomes. The trend and correlation were explored at post, 6-month, and 12-month follow-up study points.

### Data Management

Data management protocols (data collection, cleaning, and principles of analysis), research guides, and database templates were prepared at the start of the study and made available to the United Kingdom, Ghana, and Latin America teams through SharePoint. Secure research data storage was provided by the University of Southampton. Data were collected face-to-face and online by the 3 teams. The dataset collected from each team went through an independent cleaning process, and they were then merged into a combined dataset.

### Statistical Analysis

Statistical analysis was performed on the quantitative data from the assessments and questionnaires. Assessment scores (out of 32) were converted into percentages. Questions from the questionnaires where participants needed to rate a series of statements using a Likert scale (1 to 5) regarding their current or improved level of knowledge, understanding, and confidence toward the malnutrition topics were aggregated and averaged to represent their overall response in the corresponding area. Cronbach alpha was used to measure their internal consistency, and the values ranged from .88 to .92.

Summary statistics were presented based on the types of variables. Continuous variables and the differences between paired continuous variables were assessed for normality using histograms. Paired sample *t* tests were performed when comparing the pre-, post-, and 6-month assessment scores. Complete-case analysis was also performed when assessing the changes over time across all 3 assessments. Subgroup analyses were performed by country, profession, and institution type. Spearman rank correlation was used to assess the relation between knowledge gain and improved confidence. Observations with missing data in the relevant variables under investigation were automatically discarded during the above analyses. Statistical significance was set at 5%. SPSS Statistics for Windows, Version 24.0 (IBM Corp, Armonk, NY) was used to perform these analyses.

### Qualitative Analysis

Qualitative data from open-ended items in the questionnaires, interviews, and focus groups were analyzed using thematic analysis [55]. Transcripts were read and reread to aid familiarity with the data, and then data were coded to identify noteworthy findings. Codes were collated into categories to represent the dataset as a whole. Cohen kappa [56] was used to measure reproducibility across different coders (SC and APu).

## Results

### Participants

Of 1261 participants, 72.56% (915/1261) were from institutions in Ghana, 11.26% (142/1261) from Latin America, and 16.18% (204/1261) were remote online users. Table 1 shows the number of participants at each data collection point. Individual participants took part in 1 or more data collection activities.

Table 2 shows the characteristics of the participants, of whom 796 were preservice and 465 were in-service professionals. Of the in-service professionals, the majority (86.3%, 340/394) had regular close involvement with SAM children, but fewer than half of these (38.7%, 127/328) said they had received SAM training in the past. Awareness of the WHO Ten Steps was low (36.73%, 389/1059), especially among in-service professionals with only 26.3% (94/358) being aware of the guidelines. Only 20.7% (40/193) in-service professionals had received training about the WHO Ten Steps.

Access to a computer or laptop at home or at work was 100% (142/142) and 77.6% (672/866), respectively, among participants in Latin America and Ghana.

### Knowledge Gain From Training and Retention at 6 Months

Table 3 shows the gain in knowledge posttraining. Of 1261 participants, 606 took both the pre- and postassessments. The gain in knowledge was compared in relation with country, profession, and extent to which participants had completed the course. The overall mean score gain in knowledge was 11.8 (*P*<.001)—a relative increase of 41.5%. Considering only those who completed the course, the mean gain was 14 points with a relative increase of 47.8%.

**Table 1:** Number of participants at each data collection point.

Methods^a^		Number of participants			
		Prestudy^b^	Poststudy^c^	6-month follow-up^d^	12-month follow-up^e^
**Assessment**					
	Ghana	864	539	464	—
	Latin America	141	60	109	—
	Remote learning group	181	35	—	—
**Questionnaire**					
	Ghana	895	548	447	249
	Latin America	142	88	101	100
	Remote learning group	100	16	—	5
**Interview**					
	Ghana	33	14	22	19
	Latin America	4	0	14	4
	Remote learning group	4	1	—	0
**Focus group**					
	Latin America	59^f^	—	—	14^g^

^a^Participant numbers overlap between data collection methods.

^b^June to September 2015.

^c^July to October 2015.

^d^February to April 2016.

^e^July to October 2016.

^f^13 groups.

^g^7 groups.

Of those who reported prior training in SAM management (Table 2), 28.1% (101/359) achieved the preassessment pass mark compared with 13.2% (89/672) who reported no prior training. Of those who reported following the WHO guidelines in their work, 57% (26/46) achieved the preassessment pass mark. Overall, the proportion of pre- and postassessment participants who achieved scores above the pass mark posttraining was 58.7% (356/606), compared with 18.2% (110/606) pretraining. Close to two-thirds (65.5%, 271/414) of the participants who completed the course obtained the pass mark.

**Figure 3:**
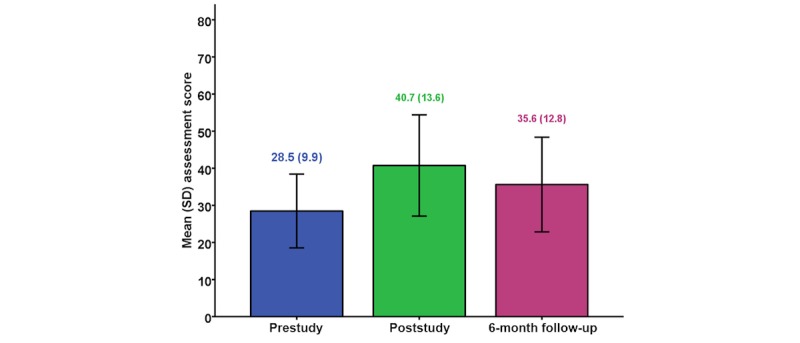
Mean (SD) assessment scores pre, post, and 6 months for the 332 participants who had all 3 assessments.

Retention of gained knowledge at 6 months is shown in Figure 3 for the 332 participants who participated in all 3 assessments. Although overall there was some loss of knowledge by 6 months, the participants’ knowledge remained significantly higher than pretraining (mean difference=7.1, 95% CI 5.9-8.4; *P*<.001) and the loss between posttraining and 6 months varied depending on whether or not participants applied their knowledge in clinical practice. For example, in-service participants who applied their knowledge retained 66% of the gained knowledge (mean scores for pre, post, and 6 months are 28.5, 46.8, and 40.5, respectively; retained knowledge=12.1/18.4) compared with 39% (mean scores for pre, post, and 6 months are 29.3, 42.2, and 34.3, respectively; retained knowledge=5.0/12.9) among those who did not apply their knowledge.

### Knowledge Application and Changes in Clinical Practice

Participants were asked to report whether they had applied the knowledge, and 85.9% of in-service professionals who took the course (128/149) reported to have applied the gained knowledge in practice. At 6 months and 12 months, 256 (51.8%, 256/494) and 143 (68.8%, 143/208) participants reported to have applied knowledge in practice and provided 528 and 366 accounts of where they applied it, respectively (up to 3 accounts per respondent). All nutritionists reported applying knowledge in their practice, with the next highest group being nurses (88%, 37/42). Table 4 presents the rankings of the main areas of application. The rankings were similar at the 2 periods, and the most common applications were related to identification and treatment of SAM.

Detailed information around changes in clinical practice was sought at 12 months from in-service and graduate preservice participants. Of 130 respondents, 115 (88.5%, 115/130) stated that they had changed their practice in line with the WHO guidelines. The reported changes are summarized in Multimedia Appendix 3.

**Table 2:** Demographics of individual participants.

Variable^a^		Ghana (N=915), n (%)	Latin America (N=142), n (%)	Remote learning group (N=204), n (%)	Total (N=1261), n (%)
**Profession**					
	Preservice (student)	597 (65.2)	106 (74.6)	93 (45.6)	796 (63.12)
	Medical doctor	4 (0.4)	28 (19.7)	12 (5.9)	44 (3.49)
	Nurse and midwife	228 (24.9)	—	8 (3.9)	236 (18.72)
	Nutritionist	21 (2.3)	8 (5.6)	16 (7.8)	45 (3.57)
	Public health	25 (2.7)	—	12 (5.9)	37 (2.93)
	Other	40 (4.4)	—	63 (30.9)	103 (8.17)
**Health care and academic institutions**					
	Hospital-based	186 (20.3)	28 (19.7)	—	214 (16.97)
	Community-based	132 (14.4)	8 (5.6)	—	140 (11.10)
	Universities	213 (23.3)	106 (74.6)	—	319 (25.30)
	Training colleges	384 (42.0)	—	—	384 (30.45)
	Remote learning group	—	—	204 (100)	204 (16.18)
**Regular close involvement with SAM^b^ children (in-service only)**					
	Yes	265 (86.9)	34 (94.4)	41 (77.4)	340 (86.3)
	No	40 (13.1)	2 (5.6)	12 (22.6)	54 (13.7)
**Aware of the WHO^c^ Ten Steps for management of SAM**					
	Yes	287 (35.0)	50 (35.5)	52 (53.6)	389 (36.73)
	No	534 (65.0)	91 (64.5)	45 (46.4)	670 (63.27)
**Received training in the management of SAM in the past**					
	Yes	294 (34.2)	61 (43.0)	30 (30.6)	385 (35.03)
	No	565 (65.8)	81 (57.0)	68 (69.4)	714 (64.97)
**Received training about the WHO Ten Steps**					
	Yes	107 (24.2)	32 (53.3)	14 (30.4)	153 (27.9)
	No	335 (75.8)	28 (46.7)	32 (69.6)	395 (72.1)
**Following the WHO Ten Steps at work (in-service only)**					
	Yes	33 (28.2)	6 (35.3)	15 (71.4)	54 (34.8)
	No	84 (71.8)	11 (64.7)	6 (28.6)	101 (65.2)

^a^Totals do not always add up to the number of participants as some questions were not answered by all.

^b^SAM: severe acute malnutrition.

^c^WHO: World Health Organization.

**Table 3:** Gain in knowledge post- versus preassessments.

Variable		Total participants (N)	Pre, mean (SD)	Post, mean (SD)	Post-pre difference	
					Mean (95% CI)	*P* value
Overall		606	28.4 (10.7)	40.2 (13.7)	11.8 (10.8 to 12.9)	<.001
**Country**						
	Ghana	512	27.0 (9.9)	39.8 (13.4)	12.8 (11.7 to 13.9)	<.001
	Latin America	60	32.6 (8.0)	42.4 (12.2)	9.7 (6.5 to 13.0)	<.001
	Remote learning group	34	41.1 (15.1)	42.6 (19.5)	1.5 (−4.7 to 7.6)	.63
	Total participants	606	28.4 (10.7)	40.2 (13.7)	11.8 (10.8 to 12.9)	<.001
**Profession**						
	Preservice (student)	316	28.7 (10.1)	37.4 (14.1)	8.7 (7.2 to 10.2)	<.001
	Medical doctor	6	44.3 (16.4)	52.1 (15.1)	7.8 (−2.8 to 18.4)	.12
	Nurse and midwife	200	24.8 (8.2)	40.8 (10.6)	16.0 (14.5 to 17.6)	<.001
	Nutritionist	21	41.1 (11.7)	55.4 (13.7)	14.3 (7.6 to 21.0)	<.001
	Public health	22	31.7 (8.9)	45.5 (14.3)	13.8 (7.9 to 19.6)	<.001
	Other	41	33.0 (14.8)	46.5 (14.5)	13.5 (9.5 to 17.5)	<.001
	Total participants	606	28.4 (10.7)	40.2 (13.7)	11.8 (10.8 to 12.9)	<.001
**Malnutrition eLearning use during the training**						
	Complete	397	29.1 (10.1)	43.0 (12.5)	14.0 (12.7 to 15.2)	<.001
	In progress	86	27.8 (11.5)	36.6 (15.0)	8.8 (5.9 to 11.6)	<.001
	Incomplete and stopped	82	22.7 (7.3)	32.1 (11.3)	9.3 (6.7 to 12.0)	<.001
	Total participants	565^a^	27.9 (10.2)	40.4 (13.5)	12.5 (11.4 to 13.6)	<.001

^a^A total of 41 participants did not respond to this question during the postintervention stage when asked if they had used the malnutrition eLearning during the training.

**Table 4:** Rankings of knowledge application reported by participants at 6 months and 12 months.

Category	Description	6 months (N=256)^a^	12 months (N=143)^a^
Identification of severe malnutrition	Using indicators (midupper arm circumference, weight-for-height) and clinical signs to assess and classify malnutrition	1	1
Treatment and management	Following the WHO^b^ Ten Steps to treat children for severe acute malnutrition	2	2
Examining for clinical signs	Examining children for signs of malnutrition and associated conditions	3	4
Taking measurements	Taking weight, height, length, midupper arm circumference correctly	4	3
Screening for malnutrition	Screening as part of growth monitoring, home visits and outreach programs, and at outpatient department and wards.	5	7
Counseling mothers	Counseling about hygiene, feeding, causes and signs of malnutrition, and how to prevent malnutrition.	6	5
Admission criteria and management option	Applying WHO criteria for admission and deciding whether admission should be to inpatient or community-based care	7	8
Other	Training other health professionals, promoting WHO guidelines, supervision, and teaching family and friends	8	6

^a^Respondents were asked to state important ways (up to 3) in which they had applied their new knowledge. Only 1 account/category was counted for each respondent. Of the 528 accounts, 449 were counted at 6 months and 327 of 366 accounts were counted at 12 months.

^b^WHO: World Health Organization.

**Table 5:** Policy and operational changes reported by in-service participants in Ghana.

Variable		In-service^a^	
		Hospital-based	Community-based
**Any policy/operational changes at work place, N**		32	27
	Yes, n (%)	27 (84)	19 (70)
**Areas of policy/operational changes, N**		27	19
	We now actively identify (screen for) malnutrition cases; n (%)	21 (78)	13 (68)
	We now diagnose SAM^b^and record in admission and discharge book; n (%)	19 (70)	6 (32)
	We now treat SAM cases; n (%)	24 (89)	12 (63)
	Non-nutritionists are now able to prepare feeds for children with SAM and do not have to wait for the nutritionist; n (%)	17 (63)	7 (37)
	The health facility has provided equipment such as scales, tape measures to enable us to measure children; n (%)	20 (74)	3 (16)

^a^Participant groups at baseline were used for data analysis, and some participants’ workplaces may have changed in the follow-up period.

^b^SAM: severe acute malnutrition.

Table 5 presents a summary of operational and policy changes at the participating hospitals and community centers, reported by individual health professionals in Ghana at 12 months. The 2 areas where change was most frequent were in active identification of malnutrition cases at community centers and hospitals and the initiation of treating SAM cases with 2 hospitals in Ghana establishing malnutrition units in the follow-up period.

### Changes in Perception

At the 6-month follow-up, participants were asked if there was any change in how they viewed malnutrition and its management as a result of taking the malnutrition eLearning course and if yes, to describe the (most significant) change. Of the 461 participants who responded to the question, 304 said yes and 282 gave a brief description of the change. Table 6 presents a summary of the participants’ responses. The most commonly reported change pertained to case-management.

### Knowledge Gain, Application, and Confidence in Patient Care Over Time

**Figure 4:**
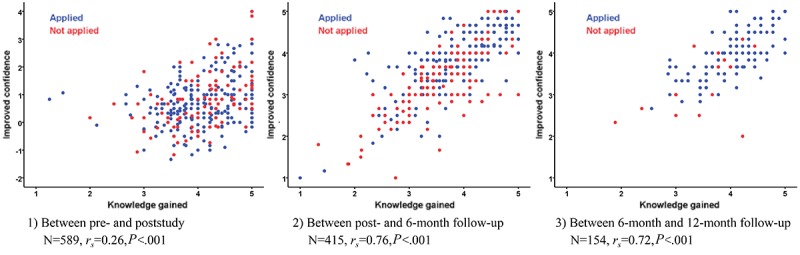
The correlation between gain in knowledge and confidence in patient care over time. Improved confidence (y-axis) in plot 1 is calculated by subtracting level of confidence at poststudy from level of confidence at prestudy, and the one in plots 2 and 3 is improved confidence between 2 corresponding time points reported.

The relation between participants’ gain in knowledge, application of knowledge, and confidence in the management of SAM over time was assessed and presented using Spearman rank correlation coefficient (*r*_*s*_). Figure 4 shows that immediately posttraining, the correlation between the gain in knowledge and confidence was weak, as we had hypothesized. In contrast, a strong correlation emerged at the 6-month follow-up and remained strong at 12 months. The areas where confidence appeared to be most widely strengthened were related to screening children for malnutrition, taking anthropometric measurements, clinical examinations, choosing the correct management option, and managing SAM children using the WHO Ten Steps. These match the areas where the participants applied gained knowledge in patient care (Table 4). Factors that participants reported to have helped improve their knowledge and enable them to apply it in their work were the visual examples in the course, step-by-step descriptions, and practical tasks, all of which made it easier to embrace and follow the concepts of malnutrition and its treatment. Of particular relevance for helping participants gain confidence was the application of the knowledge gained and its use in hands-on experience following completion of the course. The relation between knowledge gain and confidence between 6-month and 12-month follow-up was stronger among the participants who applied knowledge than the ones who did not (applied: N=127, *r*_*s*_=.74, *P*<.001 vs not applied: N=18, *r*_*s*_=.52, *P*=.03). Important constraining factors for improving confidence were mostly related to the lack of opportunity to apply new knowledge after completion of the course. This appeared especially relevant in institutions where treatment was seen as being mainly the responsibility of doctors.

**Table 6:** Summary of the reported changes in participants’ perception about malnutrition and its management.

Category	Description and example quotes	Total (N=282), n (%)
Perception about malnutrition	Changes in views and perceptions about causes of malnutrition *At first I saw malnutrition as either a curse or it happens to children of parents with low socio-economic background but now I see it in a different light.* [Nutrition trainee, Ghana] *My perception was some child was born with some malnutrition diseases like marasmus. But it has changed because I have learnt in e-learning that is not true.* [Nurse trainee, Ghana] *My perception about SAM has changed greatly and this has increased my knowledge on the causes and prevention of SAM.* [Nurse trainee, Ghana]	55 (19.5)
Assessment of malnutrition	Perception change about physiological and visual characteristics, assessment, and diagnosis of malnutrition *There are visible characteristics that before [we] did not take into account for diagnosing malnutrition.* [Nutrition graduate, Guatemala] *Previously I thought it was only children with severe muscle wasting that were malnourished but I now know that oedematous children are equally malnourished.* [Nurse trainee, Ghana] *It has helped me to differentiate between chronic and acute malnutrition and the signs and symptoms associated with them.* [Principal midwifery officer, Ghana]	51 (18.1)
Management and treatment of SAM	Change in participants’ understanding about how and where to treat children with SAM^a^ *In relation to indicators and treatment options, that is, if hospital or community.* [Nutrition graduate, El Salvador] *Initially I thought that when stabilize the patient you can just send them home but later I learnt that we have stages. We have the stabilization phase, rehabilitation and recuperation stages, so it changed.* [Health professional, Ghana] *Not all malnourished children should be treated as inpatient. First you need to classify before you start treatment for either in-patient or outpatient.* [Nutrition trainee, Ghana]	127 (45.0)
Professional roles	Perception change about participants’ professional role in the management of SAM *Well at first I thought it was only the medical officers who were supposed to treat the medical complications e.g. hypothermia and others but [now] I realize that I could also do it. If not, I could give the instruction for someone to also do it.* [Health professional, Ghana] *First when I go out to work and I see those children, I do not always want to bring them close but with the e-learning I got to know that they also need love and also education.* [Nurse, Ghana]	26 (9.2)
Other	Importance of educating mothers; self-confidence in the management of SAM and eLearning use; views about eLearning *...through the e-learning course I have come to understand that if the mother or caretaker is not well counselled on how to care, prevent and feed the child well, the child’s health will not improve.* [Community health worker, Ghana] *E-Learning will help equip nurses and all individuals including myself with the requisite knowledge to detect, manage, educate and treat all SAM cases as I have gained knowledge*. [Nurse trainee, Ghana]	23 (8.2)

^a^SAM: severe acute malnutrition.

## Discussion

### Principal Findings

The goal of the malnutrition eLearning project was to develop an innovative training solution to scale up the training of health professionals in the management of malnutrition. This evaluation study aimed to investigate whether eLearning, designed and developed appropriately, can be an effective means to train health professionals at scale who otherwise may not have the opportunity to receive relevant training. The study has shown that the malnutrition eLearning course is effective in improving the knowledge, understanding, and skills of health professionals in the diagnosis and management of children with SAM, and that the gained knowledge and skills are of practical benefit, enabling health professionals to apply them in their work. Before the training, only 26.3% (94/358) of in-service participants were aware of the WHO management guidelines despite 86.3% (340/394) having regular close involvement in the care of children with SAM. After the training, 85.9% of in-service participants who took the course (128/149) reported applying their new knowledge in their clinical practice, training colleagues who had not participated in the course, and counseling mothers.

The diverse locations and range of health professionals are a unique aspect of our study as most other investigations have been confined to 1 location and a single health profession [57]. For knowledge gained immediately after training and retained knowledge at 6 months, the findings were similar across all contexts. The same findings were observed across different professions. The participants, whether doctors, nutritionists, nurses, or students (preservice), demonstrated that the course enabled them to gain the knowledge, understanding, and skills required for the effective management of SAM. Furthermore, those who had the opportunity to apply their gained knowledge in their daily practice demonstrated higher retained knowledge at 6-month follow-up, corresponding to the theories of learning that knowledge is actively constructed by the learner through mediated or experiential learning [29,51,53]. This appeared to be particularly evident in nurses and midwives who showed the greatest increase between assessment periods, from a relatively low base, which is in accordance with the findings of Murad et al [58].

The training with the malnutrition eLearning course was voluntary and self-directed. This affected the completion of the course, which was set at 3 weeks. At the poststudy, 70.2% (449/640) of the respondents had completed the course, 16.7% (107/640) were in-progress, and 13.1% (84/640) had stopped or not yet started. However, most participants who had partially or fully completed the course actively applied the knowledge and skills they had gained to their clinical practice. They noted opportunities to share their new knowledge with other colleagues who had not participated in the training. Of the participants, 36.8% (146/397) with continued access to the course used it after the training, often together with their colleagues. The trained participants were *empowered* with their gained knowledge and motivated to change their clinical practice applying what they learned. The most cited words in open comments and interviews regarding the most significant benefits they gained from the course were “didn’t know but now I know how to...”, and “can…” and “am able to....” Positive outcomes encouraged them to seek more opportunities to apply their knowledge with “feeling proud” and “ownership” of the outcomes. Of all the professions, this change was most evident in the nurses and midwives group in Ghana. “Being able to care for SAM children” instead of “having to wait for a nutritionist or a doctor” and “being able to request equipment” with an educated reason were strong motivators for these participants to continue to use their knowledge. Changes initiated by individuals and improved outcomes led to institutional, operational, and policy changes, in contrast to findings from other self-directed learning interventions, which are reported to be effective in the knowledge domain but not in the skills or attitudes domains [58].

Knowledge gained from the course, subsequent application of the knowledge, and experience of positive outcomes changed the perceptions of the participants (66%) about malnutrition and its management, especially in relation to their professional roles in caring for malnourished children. The majority were those who did not have prior training in SAM. As we hypothesized, the relation between gain in knowledge and confidence in patient care was weak immediately after the training but strengthened over time through application of knowledge and experience of positive outcomes, similar to the result observed in an eLearning intervention to improve adherence to guidelines [59]. Learning is not just about increasing one’s knowledge, but it is about understanding, seeing things differently, and changing as a person [22-25,60]. The latter aspects of learning are what lead to behavior change, and therefore, they are particularly important to consider when designing and implementing training interventions. The observed changes among the participants suggest that the malnutrition eLearning course and subsequent application of gained knowledge in clinical practice have promoted higher levels of learning, actively advocating the WHO guideline to others, and seeking policy and operational-level changes to improve patient care as well as following the guideline by themselves.

### Relevance to Capacity Building

Severe malnutrition in childhood is a complex clinical condition that is challenging to manage but eminently preventable at community level. Under the aegis of WHO and United Nations Children’s Fund (UNICEF), the international health community has become highly skilled at effective intervention for prevention and treatment, especially in emergency contexts, but the problem remains a major challenge within the developmental context [1,61]. In large part, this limitation of our ability to apply what is known to be effective on a sustained basis is related to challenges in the education and training of frontline health professionals, especially those in remote, poorly resourced locations with limited support [62]. The WHO Ten Steps have been available for many years, but the treatment of SAM does not feature in the curricula of many medical and nursing schools, with the result that new graduates are ill equipped to care for children with SAM. Added to this is the dearth of tertiary education institutions in low- and middle-income countries that provide training in nutrition. This results in a shortage of trainers and a workforce where only few are competent in the management of severe malnutrition, leading to high mortality among severely ill malnourished children [5]. To address this issue, the international community has developed training materials such as the WHO training course on hospital-based care of SAM [63]. Most, however, are instructor-led, which severely limits the number of participants that can be trained. Available eLearning courses are often knowledge-based and limited to concepts, such as Nutrition in Emergencies by UNICEF [64]. A scalable solution is thus needed that helps the learner gain knowledge and skills and prepares them to improve their practice through self-directed learning.

This study demonstrates that the malnutrition eLearning course offers the opportunity to improve treatment practices of health professionals at scale and to make a significant contribution to building capacity for the care of children with or at risk of severe malnutrition. The internet promotes development through inclusion, efficiency, and innovation and offers great potential to help reduce inequality in training opportunities between developed and developing countries [65]. Where face-to-face training is available, health professionals should take advantage of it. Where face-to-face training is limited, eLearning can provide a useful foundation, freeing-up trainers to spend time on supervisory and support activities.

In this study, we report the process through which participants gained knowledge and their perceptions of how this helped them to change their practice, thereby leading to greater confidence in their abilities to make better-informed judgments and critically reflect upon care options. This learning process may be unremarkable within a well-structured learning environment but is particularly challenging when offered remotely in the context of severely limited resources, where failure in delivering a secure service is not unusual. In a separate paper, we will be reporting that mortality rates among severely malnourished children declined where hospital staff took the malnutrition eLearning course.

### Limitations

In this study, we sought to objectively assess the likely benefit of the malnutrition eLearning course with the target groups in locations where the issue of caring for malnourished children is a usual part of the delivery of clinical and health services but the opportunity for training is limited. This in itself is a challenge as the design of a study to achieve these ends is problematic in its execution especially among communities where research is an unusual experience and the imperative of delivering a service is a priority. Therefore, we adopted a pragmatic approach in which participation was voluntary, but the research activities and the execution of those activities were ordered. To obtain some indication of the putative generalizability of the study findings, we included a group of people who chose to take the malnutrition eLearning course for their own reasons online and sought to capture their experience using similar methods.

A weakness of the study is the lower participation rate at 6 months and 12 months. This was largely because in-service professionals who were not on duty or were working in their communities, and the preservice students who were away on placements were unable to take part. Despite the losses, sample sizes were still large enough to show significant differences and the losses were unlikely to create bias. No assessments of remote online users were made at 6 months and 12 months, and they seem to differ from the rest of the subset by having higher baseline scores. We think this might be the result of bias with online users participating in the preassessment because they wanted to test their existing knowledge, rather than advance it.

We chose the Ebel method to assess gain in knowledge. This test is typically used to assess competency, in which case a panel of specifically trained, expert, standard setters is employed. Our team was not equipped to assess competencies, and our study aim was to evaluate whether the malnutrition eLearning course led to quantifiable gains in knowledge and skills. An analysis of the participants’ answers to each question, however, may help future setters judge the 3 levels of difficulty utilized in this method. An analysis of answers would also be helpful to identify any aspects of the malnutrition eLearning course that were not well answered so that the questions, or course content, can be improved.

In this study, the malnutrition eLearning course took place without supporting activities. This is unlikely to be the case where the course is being used within a program for scaling up improved management of SAM. For example, in a program for in-service health professionals, one might expect to embed the course within a package of complementary activities such as supervision, job aids, mentoring, audit, and feedback. Such supporting activities would be expected to consolidate learning and result in higher knowledge scores and competency. In this study, assessment of knowledge took place under *exam style* with time limits and no access to manuals, posters, etc, which would be available in ideal workplace situations.

### Conclusions

The findings indicate that the self-directed malnutrition eLearning course was well received, and the learning acquired was associated with perceived improvement in practice. The training led to significant changes in knowledge and awareness of best practice in the management of SAM. Importantly, the training encouraged participants to become active change agents, not only changing their own clinical practice by following the WHO guidelines but also promoting changes among their colleagues and caregivers. At some hospitals, the changes made by individuals and associated outcomes led to operational and policy changes including the opening of 2 malnutrition units. Access to IT and internet no longer proved to be a barrier to training.

The approach taken for the capacity development of health professionals in this study can be replicated across different regions and countries; therefore, it should be considered a valuable tool in scaling up the capacity of health professionals in the management of malnourished children. We suggest that the application of good quality eLearning at scale will result in improved knowledge in the treatment and management of SAM, thus providing a scalable mechanism to train high volumes of health professionals and reducing the prevalence and impact of SAM globally.

## Appendix

Multimedia Appendix 1 Malnutrition eLearning-background and goal.

Multimedia Appendix 2 Results and findings from a field test in Uganda.

Multimedia Appendix 3 Reported changes made in clinical practice by individuals at 12-month follow-up.

## Abbreviations

IT: information technology
SAM: severe acute malnutrition
UNICEF: United Nations Children’s Fund
WHO: World Health Organization
