# Supplementary material for: Effectiveness of the Malnutrition eLearning Course for Global Capacity Building in the Management of Malnutrition: Cross-Country Interrupted Time-Series Study
Source: J Med Internet Res. 2018 Oct 3;20(10):e10396. doi: 10.2196/10396 (PMC6231886; doi:10.2196/10396)

Malnutrition eLearning

Malnutrition affects child survival

Malnutrition contributes to more than 50 percent of deaths in children under five, so reducing malnutrition is vital in child survival strategies. Most child deaths occur in South Asia and Sub-Saharan Africa, so efforts in these regions are especially important. HIV/AIDS and humanitarian emergencies aggravate the problem of severe malnutrition. Although there are as many deaths from the potentiating effects of malnutrition as from AIDS, tuberculosis and malaria (6 million/year), malnutrition fails to receive the attention it warrants in health policies and resource allocation.

The need for capacity building in the management of Severe Acute Malnutrition (SAM)

The International Malnutrition Task Force (IMTF) is an advocacy and capacity building initiative that seeks to re-instate young child malnutrition as a key focus for child survival. The Millennium Development Goal 4 seeks to reduce child mortality by two thirds. To achieve this, we need to build capacity to prevent and treat malnutrition.

Most paediatric deaths in developing countries are linked to SAM. These deaths can be prevented by following treatment guidelines. An integrated approach to prevention, timely referral, correct inpatient treatment and effective community-based care will improve child survival and development, as well as build health worker capacity and strengthen health systems.

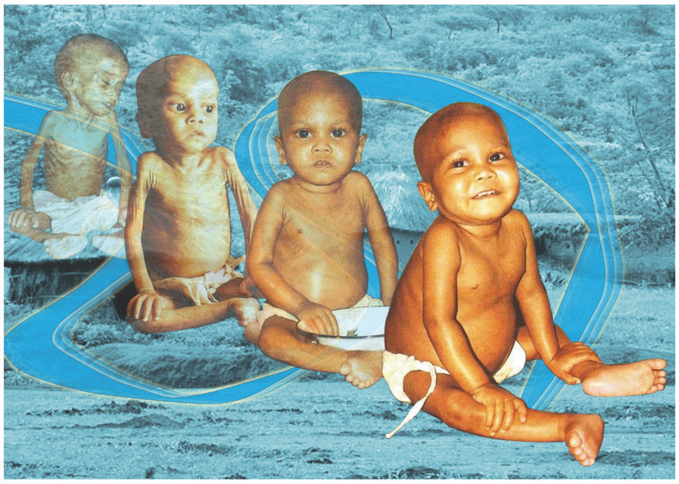

Why eLearning

Interactive learning has many benefits. It provides easy access to the materials and flexibility to learners widening their opportunity to learn. Secondly, with interactive activities it supports the learners to acquire knowledge and develop skills and competencies. And lastly, it facilitates reflective learning with interactive problem solving and reflective tasks. Face-to-face training on the management of SAM has been provided to health workers. But access is limited. eLearning can make it possible to provide a wider access to this training to many of our health workers who care daily for severely malnourished children. Furthermore, effective use of underlying educational theories and technologies in the design and delivery of the materials can enhance the learning experience.

Aims of the eLearning course

This eLearning course has been developed to provide health care professionals, who have minimal competency, with interactive learning materials that will help them to acquire knowledge and to develop skills and competencies in the care and management of infants and children with SAM, with or without complications. We aim that as a result of using the course, the health care professionals appreciate the importance of managing malnutrition appropriately and in the timely manner.

To achieve this, the course is designed to cater individualised learning for health professionals who have any responsibility for child care, especially paediatricians, nurses, medical students and nursing students.

The eLearning course

The eLearning course consists of three modules. In module 1 we discuss why SAM children need special care by explaining the visible and invisible changes that take place in malnourished children and the implications for care. Module 2 illustrates how to assess children for SAM. This includes how to make measurements, the indices to use and how and when to use them, and how to examine the child for clinical signs of SAM. Module 3 describes the WHO's 10 steps to the management of malnutrition. The integrated approach to the management recommended, ensures timely detection of cases in the community and the timely referral of those with complications that require stabilisation.

Expectation

It is expected that on completion of this course the learner will have developed basic competencies to assess children for SAM, to identify children with SAM and to manage children with SAM.

Who we are

The University of Southampton has an eLearning initiative for nutrition education, which seeks to develop the capacity in the existing and future health care workforce by enhancing their knowledge, skills and competency. The malnutrition eLearning course has been developed by the project team in collaboration with IMTF and the Royal College of Paediatrics.

For further information regarding this project, please contact Dr Sunhea Choi (s.choi@southampton.ac.uk).

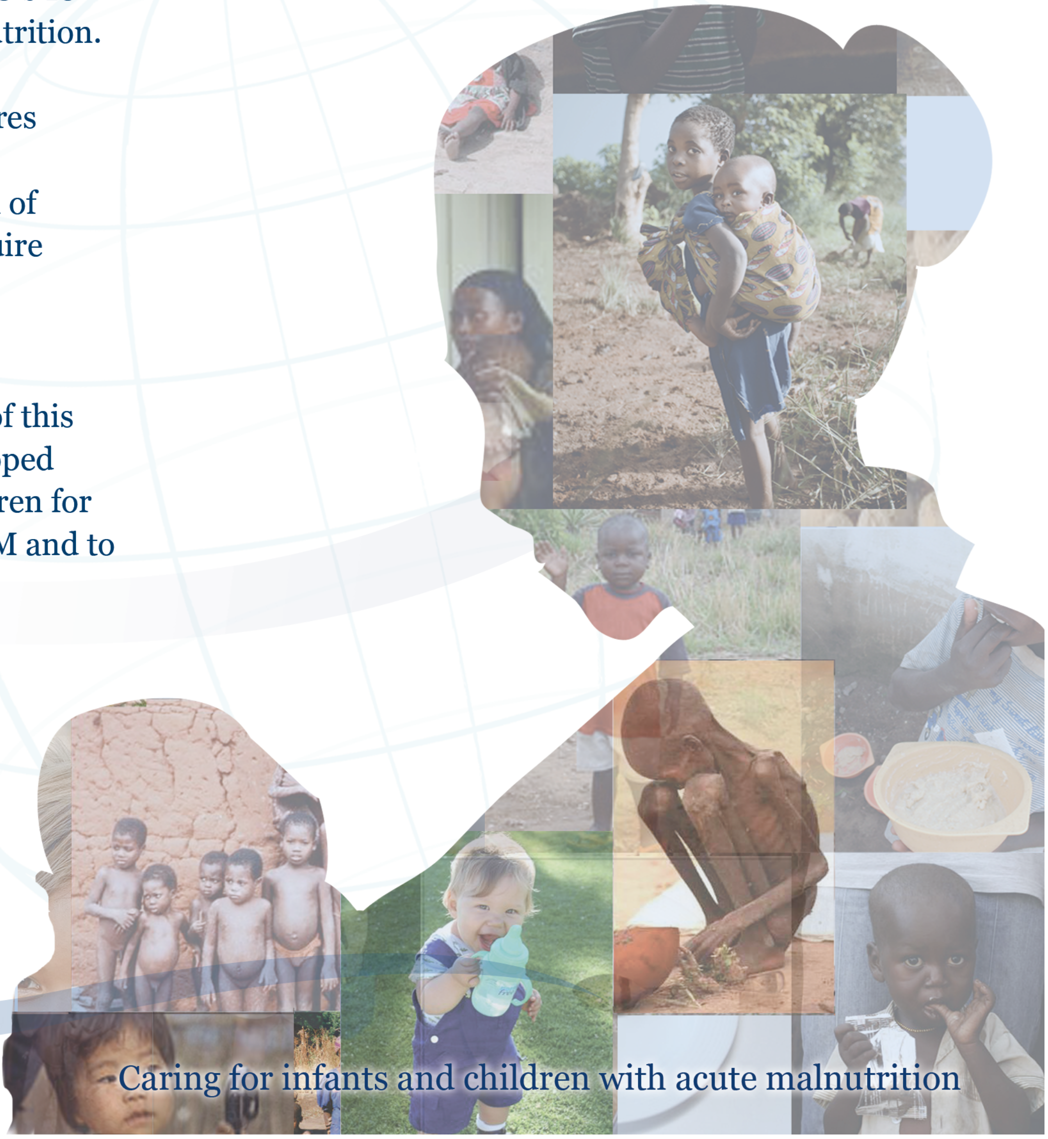

Supplement: Multimedia Appendix 1 [file jmir_v20i10e10396_app1.pdf]
