# Supplementary figures and images for: Effectiveness of the Malnutrition eLearning Course for Global Capacity Building in the Management of Malnutrition: Cross-Country Interrupted Time-Series Study
Source: J Med Internet Res. 2018 Oct 3;20(10):e10396. doi: 10.2196/10396 (PMC6231886; doi:10.2196/10396)

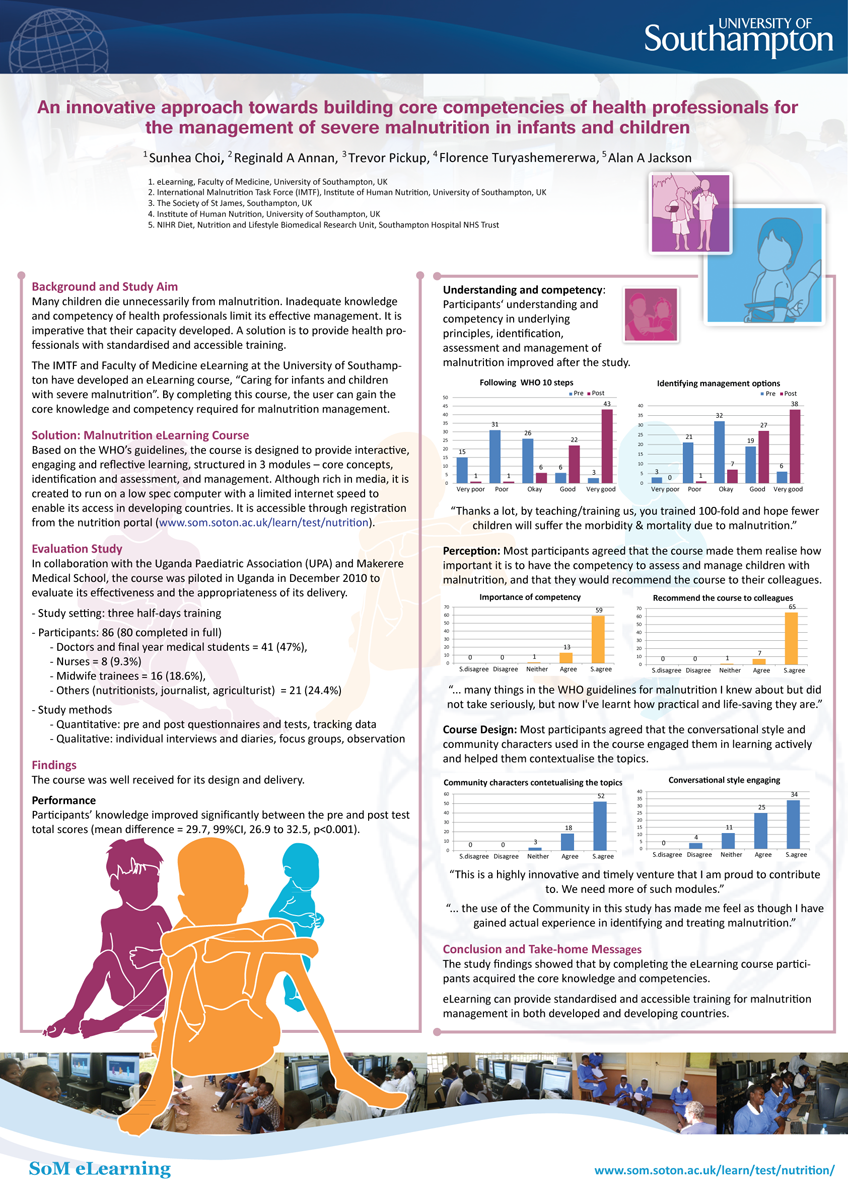

Supplement: Multimedia Appendix 2 [file jmir_v20i10e10396_app2.png]
