# Supplementary material for: Effectiveness of the Malnutrition eLearning Course for Global Capacity Building in the Management of Malnutrition: Cross-Country Interrupted Time-Series Study
Source: J Med Internet Res. 2018 Oct 3;20(10):e10396. doi: 10.2196/10396 (PMC6231886; doi:10.2196/10396)
Supplement: Multimedia Appendix 3 [file jmir_v20i10e10396_app3.pdf]

Multimedia Appendix 3. Reported changes made in clinical practice by individuals at 12-month follow-up

Changes made in clinical practice by individuals	Total (N=115), n (%)	Summary of the changes before and after training and quotes
Screening children for malnutrition	82 (65.6)	**Before:** inadequate screening with poor and inadequate techniques due to lack of knowledge about malnutrition and its identification<br>**After**: increased, improved and widened screening with correct use of indicators and clinical signs and symptoms.<br><br>**From**: "*Prior to training, using the illustrations of weight, height approximately*" **to**: "*now through electronic aids, applications, etc., always try to sift and calculate indicators more accurately*." [Doctor, Colombia]<br><br>**From**: "*Done by nutritionist*" **to**: "*Do in general during clinical outreach*" [Nurse, Ghana]<br><br>**From**: "*I only used weight and physical appearance*" **to**: "*I use the weight, height, check oedema, do appetite test and check MUAC*" [Health professional, Ghana]<br><br>**From**: *OPD, passing the appetite test*" **to**: "*Community based screening*" [Nurse, Ghana]
Using appropriate indicators, i.e. Weight-for-Height/Length and MUAC to assess malnutrition	64 (50.4)	**Before**: little or incorrect use of indicators due to lack of understanding about what the indicators were for and how to use them<br>**After**: increased, correct use of indicators, and producing equipment<br><br>**From:** "*I not know how to use MUAC neither anthropometric measures*" **to:** "*Now I do*" [Doctor, Colombia]<br><br>**From:** "*Inability to calculate according to standard.*" **to:** "*Ability to detect cases now*."[Technical officer, Ghana]<br><br>**From:** "*MUAC not available*" **to:** "*Procurement of MUAC for assessment*" [District Director of Health Services, Ghana]
Classifying the severity of malnutrition	57 (46.3)	**Before**: cases missed or misclassified due to no or limited knowledge about different types of malnutrition and how to classify the severity.<br>**After**: accurately identified cases based on anthropometric measurements, clinical signs based on WHO classification.<br><br>**From:** "*not distinguished well the severity*" **to**: "*classify it with indicators, evaluation and signs*" [Nutrition graduate, Guatemala]<br><br>**From:** "*Had previously only described if malnutrition or No. Or if had kwashiorkor marasmus*." **to:** "*More precise classification according to WHO criteria. Severe acute malnutrition*." [Doctor, Colombia]<br><br>**From:** "*I only use weight for height to classify the severity*." **to:** "*I use the weight, height, check oedema, do appetite test and check MUAC.*" [Nurse, Ghana]
Examining signs and symptoms of malnutrition	79 (64.2)	**Before**: superficial and limited due to lack of understanding about clinical signs (what to look for) and how to examine for them.<br>**After**: purposeful, increased and correct examination of clinical signs<br><br>**From**: "*I checked but could not distinguish well.*" **to:** "*I distinguish well the signs and symptoms*." [Nutrition Graduate, Guatemala]<br><br>**From**: "*Signs and symptoms went unnoticed*" **to:** "*Now pays more attention to the signs and symptoms*" [Nutrition Graduate, Guatemala]<br><br>**From**: "*Determined swelling to single observation*" **to:** "*Assess edema through acupressure in precise areas*" [Doctor, Colombia]<br><br>**From**: "*I didn't check the signs.*" **to:** "*But now I do check the signs on every child that*

		comes to me." [Nurse, Ghana]
Taking measurements (height/length, weight and MUAC)	56 (43.1)	**Before**: incorrect measurements, poor techniques and inconsistent measurements due to lack of understanding of what they are for.<br>**After**: improved techniques and consistent measurements.<br><br>**From**: "*Did not know them very well*." **to:** "*I improved in the technique*." [Nutrition Graduate, Guatemala]<br><br>**From**: "*I weighed with the clothes*." **to:** "*When I take the weight, I do it with more care and with the minimum amount of clothing*." [Nutrition Graduate, Guatemala]<br><br>**From**: "*It was done by the nutritionist*." **to:** "*She is being assisted by us*." [Nurse, Ghana]<br><br>**From**: "*I only take the weight*." **to:** "*I now take the weight, height and MUAC*." [Community nurse, Ghana]
Identifying a malnutrition management option	55 (42.3)	**Before**: inappropriate management due to no or limited understanding about management option<br>**After**: appropriate management chosen<br><br>**From**: "*Had no experience*." **to:** "*Depending on the type of malnutrition*." [Nutrition Graduate, Guatemala]<br><br>**From**: "*I did not know the 10 steps of WHO*." **to:** "*It's a practical guide when I am in hospital*." [Nutrition Graduate, Guatemala]<br><br>**From**: "*I ignored the possibility of outpatient treatment*." **to:** "*Better understanding for outpatient treatment*." [Doctor, Colombia]<br><br>**From**: "*Inpatient*" **to:** "*Community based*" [Nurse, Ghana]<br><br>**From**: "*Out-patient care only*." **to:** "*Now we do both in-patient and out-patient management*." [Field technician, Ghana]<br><br>**From**: "*We discharged them to the OPD for weekly reviews."* **to:** *"We now discharge them to the CMAM where community nurses give them appropriate feeds."* [Nutritionist, Ghana]
Treating & preventing hypoglycaemia in a malnourished child	43 (33.1)	**Before**: little done due to most having no knowledge about how to prevent and treat hypoglycaemia.<br>**After**: prevention through feeding and treatment based on the WHO guideline.<br><br>**From**: *"I did not know how to treat."* **to:** *"Application in (SAM) cases."* [Nutrition Graduate, Guatemala]<br><br>**From**: *"Not taking into account these factors."* **to:** *"I learned to detect and intervene."* [Nutrition Graduate, Guatemala]<br><br>**From**: *"Followed (hospital) protocol."* **to:** *"I request start feeding as soon as possible."* [Nutritionist, Guatemala]<br><br>**From**: *"All handled in intravenously."* **to:** *"Use of oral intervention."* [Doctor, Colombia]<br><br>**From**: *"5% dextrose"* **to:** *"Treating and preventing hypoglycaemia in children"* **to***: "F75."* [Nutritionist, Ghana]
Treating & preventing hypothermia in a malnourished child	39 (30.0)	**Before**: little or poorly done.<br>**After**: more preventative control, improved treatment, multi-disciplinary care.<br><br>**From**: "*Not watched temperature*." **to:** "*I watch temperature*." [Nutritionist, Guatemala]<br><br>**From**: "Before laminated cotton used to cover the patient. **to:** "Frequent feeding even at night." [Doctor, Colombia]<br><br>**From**: "*Covering of the body with clothes*." **to:** "*Mother and baby lie in the same cot*."

		[Nutritionist, Ghana]<br>**From**: "*We(nutritionists) only gave feeds*." **to:** "*It is done together with the nurses on the ward*." [Nutritionist, Ghana]
Treat & preventing dehydration in a malnourished child	47 (36.2)	**Before**: many not knowing how to treat dehydration<br>**After**: more attention and use of ReSoMal<br>**From:** "*Signs of dehydration were not checked*" **to**: "*dehydration signs are evaluated*". [Nutrition Graduate, Guatemala]<br>**From**: "*In hospital I did not suggest the use of ReSoMal.*" **to:** "*We are managing ReSoMal*." [Nutritionist, Guatemala]<br>**From**: "*IVF R/L ORS*" **to:** "*ReSoMal*" [Nurse, Ghana]<br>**From**: "*I did not have any knowledge.*" **to:** "*I can now treat and prevent.*" [Community nurse, Ghana]<br>**From**: "*Infusion*" **to:** "*ReSoMal*" [Nutritionist, Ghana]
Treating electrolyte imbalance in a malnourished child	30 (23.1)	**Before**: little done due to lack of knowledge.<br>**After**: management of electrolyte imbalance.<br>**From**: "*I did not know how to treat electrolyte imbalance.*" **to:** "*I can treat electrolyte imbalance.*" [Nutrition Graduate, Guatemala]<br>**From**: "*Not considered to meet this aspect.*" **to:** "*The hospital is considering applying for exams.*" [Nutritionist, Guatemala]<br>**From**: "*Was not in the position to do that.*" **to:** "*Positive about managing electrolyte imbalance.*" [Nurse, Ghana]<br>**From**: "*No idea on management.*" **to:** "*Able to manage now*." [Technical officer, Ghana]
Treating & preventing infections in a malnourished child	45 (34.6)	**Before**: mostly no treatment due to lack of knowledge.<br>**After**: using antibiotics and more attention to prevent infection.<br>**From**: "*Not made.*" **to:** "*It refers to the medical center and given timely treatment.*" [Nutrition Graduate, Guatemala]<br>**From**: "*The real risks of infection were not understood even though the protocol includes antibiotics.*" **to:** "*Antibiotic starts and monitors whether the child has infection*." [Nutritionist, Guatemala]<br>**From**: "*Infection prevention sometimes not done.*" **to:** "*Infection prevention always done*." [District Director of Health Services, Ghana]<br>**From**: "*I was unable to teat and prevent infection.*" **to:** "*I can now treat and prevent infection in malnourished children*." [Community nurse, Ghana]
Treating micronutrient deficiencies in a malnourished child	54 (41.5)	**Before**: lack of or incorrect treatment due to lack of understanding.<br>**After**: initiation and correct treatment<br>**From**: "*I did not know what micronutrients to supplement.*" **to:** "*Now I do.*" [Nutrition Graduate, Guatemala]<br>**From**: "*I did not know micronutrient deficiency in malnourished children.*" **to:** "*Now I know how the deficiencies should be handled.*" [Nutritionist, Guatemala]<br>**From**: "*Previously, use of iron from the start. Transfusions of red blood cells in more liberal form.*" **to:** "*Iron in the maintenance phase. Transfusion only if accurate indication.*"[Doctor, Colombia]<br>**From**: "*Multivitamin syrup was given.*" **to:** "*Education is given to care givers to feed child with verities of fruits.*" [Nutritionist, Ghana]

Feeding a malnourished child cautiously at the start of treatment	58 (44.6)	**Before**: no cautious feeding due to lack of understanding about what it was and how to do.<br>**After**: cautious feeding initiated and done correctly. Feeding in rehabilitation phase too improved.<br><br>**From**: *"I did not know."* **to:** *"Cautious to prevent signs of intolerance*." [Nutrition Graduate, Guatemala]<br><br>**From**: *"Cautious feeding was lacking."* **to:** *"Cautious feeding done appropriately."* [District Director of Health Services, Ghana]<br><br>**From**: *"No visiting"* **to:** *"Follow ups are being done to supervise feeding*." [Nutritionist, Ghana]<br><br>**From**: *"I referred."* **to:** *"I refer and when they get back to the community I monitor both mother and child and give them the necessary assistance."* [Community nurse, Ghana]
Providing sensory stimulation to a child with malnutrition	36 (27.7)	**Before**: rarely done due to lack of understanding about its importance.<br>**After**: actively delivered – part of treatment and follow-up.<br><br>**From**: *"I gave no stimulation."* **to:** *"Providing such stimulation to children."* [Nutrition Graduate, Guatemala]<br><br>**From**: *"Exit it (case) only with medical treatment."* **to:** *"I refer to early stimulation*." [Nutritionist, Guatemala]<br><br>**From**: *"Did not consider sensory stimulation."* **to:** *"Now support calls on psychologist or educators."* [Nutritionist, Guatemala]<br><br>**From**: *"Was not done."* **to:** *"We do it now."* [District Director of Health Services, Ghana]
Teaching the parents of malnourished children how to prevent malnutrition from recurring	68 (52.3)	**Before**: no or limited education given to the parents.<br>**After**: increased, enhanced education and counselling.<br><br>**From**: *"No great importance placed on them."* **to:** *"I provide them with adequate nutrition education to each case before discharge."* [Nutrition Graduate, Guatemala]<br><br>**From**: *"Was not taught."* **to:** *"Parents are trained in health centers."* [Nutrition Graduate, Guatemala]<br><br>**From**: *"I didn't counsel them on proper feeding."* **to:** *"Now I do a lot of counselling to parents whose children are recovering*." [Nurse, Ghana]<br><br>**From**: *"I did health education."* **to:** *"I educate and do surveillance and if necessary referral of the malnourished children identified."* [Community nurse, Ghana]<br><br>**From**: *"We just counselled the parents."* **to:** *"Community nurses sit together with parents and watch them feed their children."* [Nutritionist, Ghana]
